# X-linked palindromic gene families *4930567H17Rik* and *Mageb5* are dispensable for male mouse fertility

**DOI:** 10.1038/s41598-022-12433-9

**Published:** 2022-05-20

**Authors:** Evan R. Stark-Dykema, Eden A. Dulka, Emma R. Gerlinger, Jacob L. Mueller

**Affiliations:** Department of Human Genetics, University of Michigan Medical School, Ann Arbor, MI 48109 USA

**Keywords:** Evolutionary biology, Spermatogenesis

## Abstract

Mammalian sex chromosomes are enriched for large, nearly-identical, palindromic sequences harboring genes expressed predominately in testicular germ cells. Discerning if individual palindrome-associated gene families are essential for male reproduction is difficult due to challenges in disrupting all copies of a gene family. Here we generate precise, independent, deletions to assess the reproductive roles of two X-linked palindromic gene families with spermatid-predominant expression, *4930567H17Rik* and *Mageb5*. Sequence analyses reveals mouse *4930567H17Rik* and *Mageb5* are orthologs of human HSFX3 and MAGEB5, respectively, where *4930567H17Rik/*HSFX3 is harbored in a palindrome in humans and mice, while *Mageb5* is not. Additional sequence analyses show *4930567H17Rik* and HSFX3 are rapidly diverging in rodents and primates, respectively. Mice lacking either *4930567H17Rik* or *Mageb5* gene families do not have detectable defects in male fertility, fecundity, spermatogenesis, or in gene regulation, but do show differences in sperm head morphology, suggesting a potential role in sperm function. We conclude that while all palindrome-associated gene families are not essential for male fertility, large palindromes influence the evolution of their associated gene families.

## Introduction

Large (> 8 kb) palindromes are inverted segmental duplications that contain nearly-identical (> 99%) DNA sequences. Large palindromes are enriched on the X and Y chromosomes in mammals^1,2^ and harbor gene families (≥ 2 copies of nearly-identical gene sequence) expressed predominantly during spermatogenesis^3,4^. Males are more susceptible than females to deleterious mutations in single-copy X-linked genes, because males are hemizygous (carry only one X compared to females two X chromosomes)^5^. Having a second copy of a sex-linked gene could potentially provide protection against hemizygotic susceptibility to new deleterious mutations. The presence of genes in two copies on either the X or Y in large palindromes may have evolved to provide protective roles for genes that are important for male fertility^6^. For example, in mice, three of four independent deletions of large palindrome arrays result in male infertility^7–9^. Additionally, large deletions of Y chromosome palindromes in men can result in male infertility^10,11^. These previous findings suggest genes harbored within large palindromes may be essential for male fertility.

Deletions of large palindrome arrays also remove both associated gene families and the palindrome structures, making it difficult to separate whether the loss of the gene families or palindrome structures contribute to male infertility. Unlike palindrome arrays, which contain multiple segmental duplications in each arm, singleton palindromes contain a single segmental duplication in each arm. To investigate the importance of palindrome structure, previous studies have disrupted arms of two independent singleton palindromes, carrying the X-linked gene families *4930567H17Rik* and *Mageb5*^12^. Deletion or inversion of arms within singleton palindromes did not alter overall fertility^12^. These studies did not, however, investigate whether gene families within these palindromes are necessary for male fertility since one copy of the gene family was left intact within the remaining palindrome arm. If large palindromes provide protective functions for genes that are essential for male fertility, then complete loss of singleton palindrome-associated gene families could result in defects in spermatogenesis and male fertility.

To test if singleton palindrome-associated gene families *4930567H17Rik* or *Mageb5* are important for male fertility, we independently deleted both gene copies*.* In addition to building on our prior studies^12^ on the palindrome structure of the *4930567H17Rik* and *Mageb5* gene families, we chose to independently delete both gene families because they exhibit canonical features found in other X-palindrome gene families (e.g. predominant expression in spermatids, high level of nucleotide identity between palindrome arms)^3^. Moreover, the presence of a single protein-coding gene in each palindrome enables us to more confidently ascribe associated reproductive phenotypes to loss of the deleted gene family^12^. We find both *4930567H17Rik* or *Mageb5* gene families in mice have orthologs in humans, but despite this conservation, mice lacking either gene family do not exhibit detectable defects in male fertility and post-meiotic spermatogenesis. We observe several abnormalities in sperm head morphology, indicating that while *4930567H17Rik* or *Mageb5* are not necessary for male fertility, both genes likely play a role in post-meiotic sperm development. Overall, our study supports that the *4930567H17Rik* and *Mageb5* gene families may play roles in spermatogenesis, but are not necessary for overall male fertility in C57BL/6 J mice. Our findings that *4930567H17Rik* or *Mageb5* are dispensable for male fertility is consistent with previous efforts demonstrating many single-copy testes-specific genes are also dispensable for male fertility in mice^13–15^. Our studies add to previous findings suggesting *4930567H17Rik* and *Mageb5* palindrome structures are not essential for male fertility or spermatogenesis^12^.

## Results

### Mouse *4930567H17Rik* is a highly diverged ortholog of human *HSFX3*, while mouse *Mageb5* is a conserved ortholog of human *MAGEB5*

**Figure 1:**
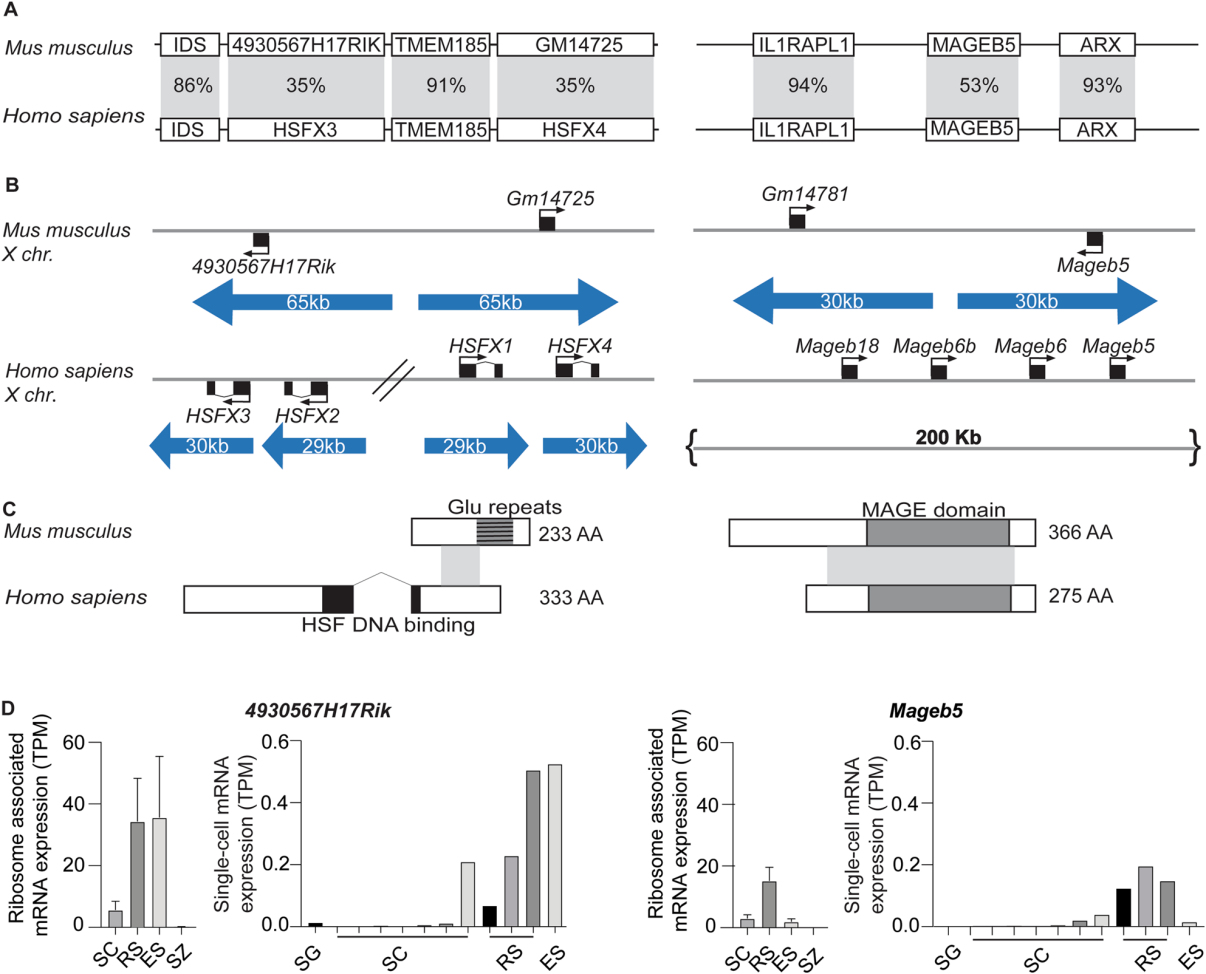
*4930567H17RIK* and *MAGEB5* share orthologs between mouse and humans. (**A**) Syntenic region of *4930567H17RIK* and *MAGEB5* between mouse and human with percent amino acid identity in the shaded region. *4930567H17Rik* shares 35% amino acid identity (across 18% of the protein) with *HSFX3. Mageb5* shares 53% amino acid identity (across 81% of the protein) with *MAGEB5* (**B**) Palindrome structure of the regions containing *4930567H17Rik, HSFX3, Mageb5*, and *MAGEB5*. Palindrome arms are represented as blue arrows. *HSFX3* is amplified compared to *4930567H17Rik*. *Mageb5* does not share palindrome structure between mouse and human. (**C**) Intron–exon structure for *4930567H17Rik*/*HSFX3*, and *MAGEB5*/MAGEB5 showing protein domains (HSF DNA binding domain (black), glutamic acid repeats (dark grey), MAGE domain (light grey)) and amino acid sequence similarity (grey shading between species). (**D**) Reanalyzed ribosome profiling and natural log values of UMI counts from single-cell RNA-seq data for *4930567H17Rik* and *Mageb5* (data sets taken from Wang, et. al and Green, et. al.^18,19^). Ribosome association and mRNA expression is most strongly seen in post meiotic cells for both *4930567H17Rik* and *Mageb5*. *SG* spermatogonia, *SC* spermatocyte, *RS* round spermatid, *ES* elongating spermatid, *SZ* spermatozoa.

X-linked gene families associated with large palindromes can have orthologs in other species or be independently acquired^4^. To assess possible orthologs of mouse *4930567H17Rik* and *Mageb5* in humans, we compared their protein sequence via BLASTP and found mouse *4930567H17Rik* is orthologous to human Heat Shock Transcription Factor X linked Member 3 (*HSFX3*) and mouse *MAGEB5* is orthologous to human MAGEB5 (Fig. 1A). We further examined whether the genomic regions between mouse and human are syntenic (i.e. if they share flanking orthologous genes). In mouse, *4930567H17Rik* is flanked by the genes *Iduronate 2-sulfatase* (*Ids*) and *Transmembrane Protein 185a* (*Tmem185a*) which also flank *HSFX3* in humans. Interleukin 1 receptor accessory protein-like 1 *(Il1RAPl1) and Aristaless related homeobox* (*Arx*) flank *Mageb5* in both human and mouse (Fig. 1A). This data supports that *4930567H17Rik* and HSFX3 and *Mageb5* and MAGEB5 are orthologous between human and mice*.*

In mouse, two gene copies of *4930567H17Rik* and *Mageb5* exist within palindromic sequences (Fig. 1B), however, the copy number of both genes is different on the human X chromosome. In humans, *HSFX3* is present in four copies (annotated as *HSFX1-4)* and *MAGEB5* is inverted in a unique non-palindromic sequence. Human *MAGEB5* has additional neighboring *MAGEB* gene family copies, but none of the gene family members are within palindromes (Fig. 1B). BLASTP alignments and synteny of *4930567H17Rik/HSFX3* and *Mageb5/MAGEB5* suggest both X-linked gene families were present in the common ancestor of mouse and humans ~ 80 million years ago (MYA), but diverged at the sequence level, as in the case of *4930567H17Rik,* or at the level of palindrome structure, as in the case of *Mageb5.*

### Mouse *4930567H17Rik* and human *HSFX3 *are rapidly diverging protein-coding genes

To further understand how *4930567H17Rik* and *HSFX3* diverged, we compared the evolutionary dynamics and intron–exon structures of *4930567H17Rik* in rodents and *HSFX3* in primates. Previous studies have shown both copies of *4930567H17Rik* (gene accession #’s NM_001033807, NM_001081476.1) exhibit rapid sequence divergence in rodents; having a K_a_/K_s_ value of 1.81 when compared across three *Mus* lineages and *R. norvegicus* as an outgroup^16^. We find primate *HSFX3* has a K_a_/K_s_ value of 1.20, indicating *4930567H17Rik* and *HSFX3* sequence is rapidly diverging in both rodents and primates. The rapid sequence divergence of *4930567H17Rik* in rodents may have been facilitated by loss of an exon. Other mammalian *HSFX3* orthologs, including human *HSFX3,* encode two exons, while *4930567H17Rik* encodes a single exon (Fig. 1C). HSFX3 has a DNA binding domain spanning the splice junction between exon 1 and 2. *4930567H17Rik* produces a predicted protein that shares amino acid identity only with HSFX3. While 4930567H17RIK lacks the DNA-binding domain, it possesses an expanded glutamic acid repeat at the C-terminus end (Fig. 1C). Interestingly, the first exon of mouse *4930567H17Rik* is pseudogenized and remnants of the first exon are found outside of the palindrome, via sequence comparisons (Fig S1). RNA-seq data supports the ancestral first exon of HSFX is not transcribed in mice and thus the second exon is the only remaining functional exon in *Mus musculus* (Fig S1).

The predicted annotation of *4930567H17Rik* is a long non-coding RNA^17^. However, we find evidence to support that *4930567H17Rik* is a protein-coding gene despite loss of an exon and rapid sequence divergence. First, *4930567H17Rik* encodes a large open reading frame (233 amino acids), conserved across rodents (Fig. 1C). Despite the rapid sequence divergence, *4930567H17Rik* has not acquired new nonsense mutations typical of pseudogenes or long non-coding RNAs. Second, reanalysis of ribosome profiling data^18^ from sorted spermatogenic cells demonstrates *4930567H17Rik* RNA is most highly associated with ribosomes in round spermatids and elongating spermatids, consistent with single cell RNA-seq studies, altogether indicating *4930567H17Rik* mRNA is likely translated (Fig. 1D). Overall, we conclude that the protein-coding sequence of *4930567H17Rik/HSFX3* is rapidly diverging across mammals, and the exon containing the HSF DNA-binding domain has been lost along the Mus lineage, after divergence with rat.

### Generation of mice lacking both copies of the palindrome associated gene families *4930567H17Rik* or *Mageb5*

**Figure 2:**
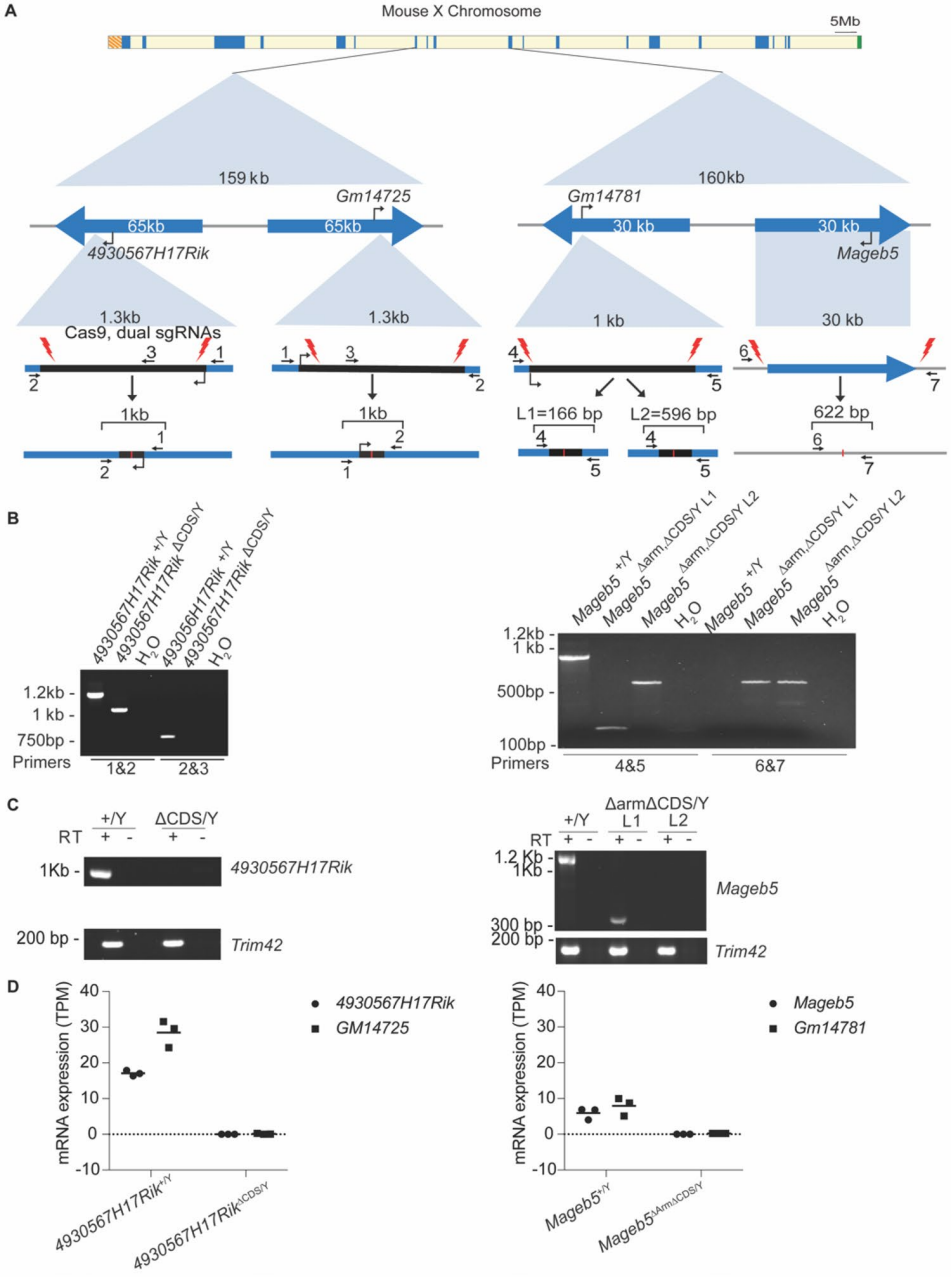
Creation of *4930567H17Rik*^∆CDS/Y^, and *Mageb5*^∆Arm∆CDS/Y^ mice. (**A**) Top: Schematic of the mouse X chromosome showing singleton palindromes (blue) the pseudoautosomal region (green, right end), and centromere (orange, left end). Middle: Diagrams of the *4930567H17Rik* and *Mageb5* palindromes showing palindrome arms as blue arrows. Bottom: Coding sequence of *4930567H17Rik* and one copy of *Mageb5* with CRISPR cut sites (red lightning bolts) and genotyping primers (black arrows with numbers). (**B**) PCR genotyping of DNA from mutant and wild type *4930567H17Rik* and *Mageb5* mice. Numbered primers correspond to panel A. (**C**) RT-PCR of *4930567H17Rik* and *Mageb5* cDNA from one 4930567H17Rik line and *Mageb5* L1 and L2. L1 shows a product due to a small portion of the transcript still being produced. (**D**) RNA-seq data for *4930567H17Rik*^∆CDS/Y^, and *Mageb5*^∆Arm∆CDS/Y L1^ mice. *GM14725* and *Gm14781* are the palindrome arm gene copies of *4930567H17Rik* and *Mageb5,* respectively. See Fig S2 for full-size images of (**B**,**C**).

To determine whether the *4930567H17Rik* and *Mageb5* gene families are necessary for male fertility, we generated complete null mutant mice for both *4930567H17Rik* and *Mageb5* by using CRISPR/Cas9 (Fig. 2A). We deleted both *4930567H17Rik* and *Gm14725* protein-coding gene copies (mm10 chrX:70,394,006:70,394,659 and chrX:70,545,638:70,546,279)*,* resulting in a null mutant mouse line (*4930567H17Rik*^∆CDS/Y^), as evidenced by RT-PCR and RNA-seq (Fig. 2C,D). For *Mageb5*, we utilized *Mageb5*^∆Arm/+^ mice, which already had one *Mageb5* palindrome arm deleted^12^. We deleted the second copy of *Mageb5,* annotated as *Gm14781,* by specifically targeting the remaining *Gm14781* protein-coding gene copy in *Mageb5*^∆Arm/+^ mice (Fig. 2A), to generate two null mutant *Mageb5*^∆Arm∆CDS/Y^ lines (L1 and L2). In L1 mice, ~ 860 bp were removed (mm10 chrX:91,634,446–91,635,305), while in L2 ~ 430 bp were removed (mm10: chrX:91,634,446–91,634,878) (Fig. 2B, right), yielding PCR products of 166 bp (L1) and 596 bp (L2). The translational start site was removed in both lines. To assess if *Mageb5*^∆Arm∆CDS/Y^ L1 and L2 produced RNA, we performed RT-PCR, followed by Sanger sequencing. We find that *Mageb5*^∆Arm∆CDS/Y^ L1 yields a highly truncated RNA product (322 bp) and L2 does not yield any detectable RNA product (Fig. 2C). Sequencing of the 322 bp cDNA from L1 reveals a small, predicted peptide (< 80 amino acids) (Fig S3)**.** RNA-seq analyses of L1 support a lack of mRNA expression for both copies of the *Mageb5* gene in *Mageb5*^∆Arm∆CDS/Y L1^ mice (Fig. 2D). Our results support the successful removal of ~ 650 bp of both copies of *4930567H17Rik* and ~ 860 bp and ~ 430 bp of *Mageb5*, and 30 Kb of a palindromic arm containing *Mageb5,* to yield *4930567H17Rik*^∆CDS/Y^ and *Mageb5*^∆Arm∆CDS/Y^ null mutant mice, respectively.

### *4930567H17Rik* and *Mageb5* do not play major transcriptional regulatory roles during mouse spermatogenesis

We tested whether mouse 4930567H17RIK or MAGEB5 protein regulates transcription, since 4930567H17RIK is orthologous to a heat shock transcription factor, HSFX3, and MAGE proteins are known to regulate transcription^20,21^. We assessed whether mice lacking *4930567H17Rik* or *Mageb5* exhibit differences in gene expression. We performed whole testis RNA-seq to identify differentially expressed genes between *4930567H17Rik*^∆CDS/Y^ or *Mageb5*^∆Arm∆CDS/Y^ mice and their wild-type littermate controls. We identified 21 and 27 differentially expressed (p-value < 0.0001) genes for *4930567H17Rik*^∆CDS/Y^ and *Mageb5*^∆Arm∆CDS/Y^ mice, respectively (Fig S4, Table S1 + S2). Consistent with our above analyses supporting a lack of *4930567H17Rik* and *Mageb5* expression, both copies of each gene family (*4930567H17Rik* and *Gm14725* and *Mageb5* and *Gm14781*) are the top two differentially expressed genes and are significantly downregulated in *4930567H17Rik*^∆CDS/Y^ and *Mageb5*^∆Arm∆CDS/Y L1^ mice (Fig S4, Table S1 + S2). *4930567H17Rik* or *Mageb5* thus influence the transcription of a limited number of genes, suggesting that they have a minimal, if any role, in regulating gene transcription during spermatogenesis.

### *4930567H17Rik*^∆CDS/Y^ and *Mageb5*^∆Arm∆CDS/Y^ mice display testis histology, sperm counts, and sperm motility similar to wild-type mice

**Figure 3:**
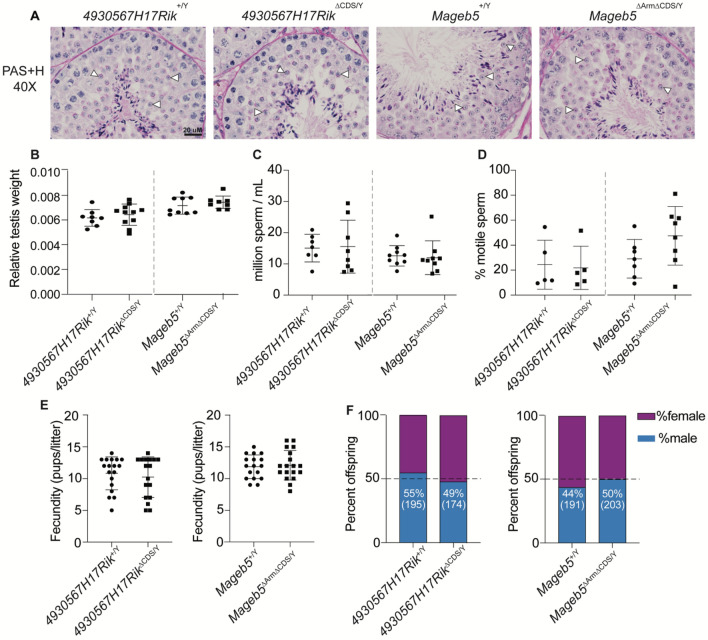
4*930567H17Rik*^∆CDS/Y^ and *Mageb5*^∆Arm∆CDS/Y^ mice display testis histology, testis size, sperm counts, and motility similar to *4930567H17Rik*^+/Y^ and *Mageb5*^+/Y^ mice. (**A**) Periodic Acid-Schiff and Hemoxylin stained sections showing normal progression of spermatogenesis in *4930567H17Rik*^∆CDS/Y^ and *Mageb5*^∆Arm∆CDS/Y^ mice. Arrows indicate presence of round spermatids. (**B**) Total testis weight normalized to total body weight (g). *4930567H17Rik*^+/Y^ n = 8, *4930567H17Rik*^∆CDS/Y^ n = 11, *Mageb5*^+/Y^ and *Mageb5*^∆Arm∆CDS/Y^ n = 9 (**C**) Sperm counts. Each point represents a single individual where 2 technical replicates were counted and averaged. *4930567H17Rik*^+/Y^ n = 7, *4930567H17Rik*^∆CDS/Y^ n = 8, *Mageb5*^+/Y^ and *Mageb5*^∆Arm∆CDS/Y^ n = 9. (**D**) Percent motile sperm. *4930567H17Rik*^+/Y^ and *4930567H17Rik*^∆CDS/Y^ n = 5, *Mageb5*^+/Y^ n = 7 and *Mageb5*^∆Arm∆CDS/Y^ n = 8. (**E**) Pups/litter from mating three males from each genotype with CD1 females. *4930567H17Rik*^+/Y^ n = 18, *4930567H17Rik*^∆CDS/Y^ and *Mageb5*^+/Y^ n = 17 and *Mageb5*^∆Arm∆CDS/Y^ n = 18 litters. (**F**) Sex genotyping performed on pups from litters shown in (**E**). Number of male offspring are shown as percentage of total number of pups in parenthesis below. All comparisons in (**B**–**F**) were performed using an unpaired two-tailed student’s t-test between *4930567H17Rik*^+/Y^ versus *4930567H17Rik*^∆CDS/Y^ and *Mageb5*^+/Y^ versus *Mageb5*^∆Arm∆CDS/Y^. All error bars represent mean with standard deviation.

To assess if *4930567H17Rik*^∆CDS/Y^ and *Mageb5*^∆Arm∆CDS/Y^ mice exhibit defects in spermatogenesis or sperm biology, we examined if *4930567H17Rik*^∆CDS/Y^ and *Mageb5*^∆Arm∆CDS/Y^ mice display defects in post-meiotic sperm development via testis histological sections. Periodic acid-shift and hematoxylin (PAS + H) stained slides of testis tubules revealed that all stages of spermatogenesis were present and apparently similar to that of wild-type controls (Fig. 3A) (Fig S5), including a normal appearance of round spermatids, the cell type in which *4930567H17Rik* and *Mageb5* are predominantly expressed^3,12^. In agreement with this finding, no significant differences in testis size (assayed via testis/body mass ratios) were detected (*4930567H17Rik*^∆CDS/Y^ = 0.006428 ± 0.00086, *4930567H17Rik*^+/Y^ = 0.006167 ± 0.00067, p = 0.47; *Mageb5*^∆Arm∆CDS/Y^ = 0.007342 ± 0.00050, *Mageb5*^+/Y^ = 0.007163 ± 0.00069, p = 0.54) (Fig. 3B). Thus, in the absence of *4930567H17Rik* and *Mageb5* gene families, sperm development proceeds similar to wild-type testes.

We also assessed whether there were defects in total sperm or sperm motility in *4930567H17Rik*^∆CDS/Y^ and *Mageb5*^∆Arm∆CDS/Y^ mice. There were no differences in the number of sperm produced between *4930567H17Rik*^+/Y^ and *4930567H17Rik*^∆CDS/Y^ and, *Mageb5*^+/Y^ and *Mageb5*^∆Arm∆CDS/Y^ mice (*4930567H17Rik*^∆CDS/Y^ = 15.6 ± 8.5 × 10^6^/ml, *4930567H17Rik*^+/Y^ = 15.0 ± 4.4 × 10^6^/ml, p = 0.88, n = 5; *Mageb5*^∆Arm∆CDS/Y^ = 11.9 ± 5.4 × 10^6^/ml, n = 8, *Mageb5*^+/Y^ = 12.6 ± 3.3 × 10^6^/ml, n = 7, p = 0.78) (Fig. 3C). Sperm from both *4930567H17Rik*^∆CDS/Y^ and *Mageb5*^∆Arm∆CDS/Y^ mice were > 50% motile (*4930567H17Rik*^∆CDS/Y^ = 60 ± 2.6%, n = 2 and *Mageb5*^+/Y^ = 67 ± 9.9% motile, n = 2). This motility was further examined via a swim-up assay and did not differ regardless of genotype (*4930567H17Rik*^∆CDS/Y^ = 22.91 ± 17.99%, *4930567H17Rik*^+/Y^ = 24.95 ± 20.37%, p = 0.87; *Mageb5*^∆Arm∆CDS/Y^ 49.28 ± 24.43%, *Mageb5*^+/Y^ = 30.14 ± 16.09%, p = 0.10) (Fig. 3D).

### *4930567H17Rik*^∆CDS/Y^ and *Mageb5*^∆Arm∆CDS/Y^ mice exhibit wild-type levels of fertility, fecundity, and sex ratio

To assess if *4930567H17Rik*^∆CDS/Y^ and *Mageb5*^∆Arm∆CDS/Y^ mice sire fewer offspring, *4930567H17Rik*^∆CDS/Y^ and *Mageb5*^∆Arm∆CDS/Y^ mice were mated to female CD1 mice. Both *4930567H17Rik*^∆CDS/Y^ and *Mageb5*^∆Arm∆CDS/Y^ mice exhibited wild-type levels of fertility and fecundity, producing litters of equivalent size to wild-type controls (*4930567H17Rik*^∆CDS/Y^ = 10.2 ± 3.2 pups/litter, n = 174, *4930567H17Rik*^+/Y^ = 10.8 ± 2.6 pups/litter, n = 195, p = 0.55; *Mageb5*^∆Arm∆CDS/Y^ = 12.1 ± 2.3 pups/litter, n = 203, *Mageb5*^+/Y^ = 11.9 ± 1.9 pups, n = 191, p = 0.77) (Fig. 3E). Previous studies have found X-palindrome-associated genes can skew the offspring sex ratio^8^, thus we genotyped the sex of all offspring. No sex ratio distortion was detected in the offspring of either *4930567H17Rik*^∆CDS/Y^ or *Mageb5*^∆Arm∆CDS/Y^ mice (*4930567H17Rik*^∆CDS/Y^ = 49% male offspring, *4930567H17Rik*^+/Y^ = 55% male offspring, p = 0.25; *Mageb5*^∆Arm∆CDS/Y^ = 50% male offspring, *Mageb5*^+/Y^ = 44% male offspring, p > 0.99, unpaired two-tailed t-test) (Fig. 3F), suggesting mouse *4930567H17Rik* or *Mageb5* do not influence sex ratio distortion, autonomously.

### *4930567H17Rik*^∆CDS/Y^ and *Mageb5*^∆Arm∆CDS/Y^ mice display altered sperm morphology

**Figure 4:**
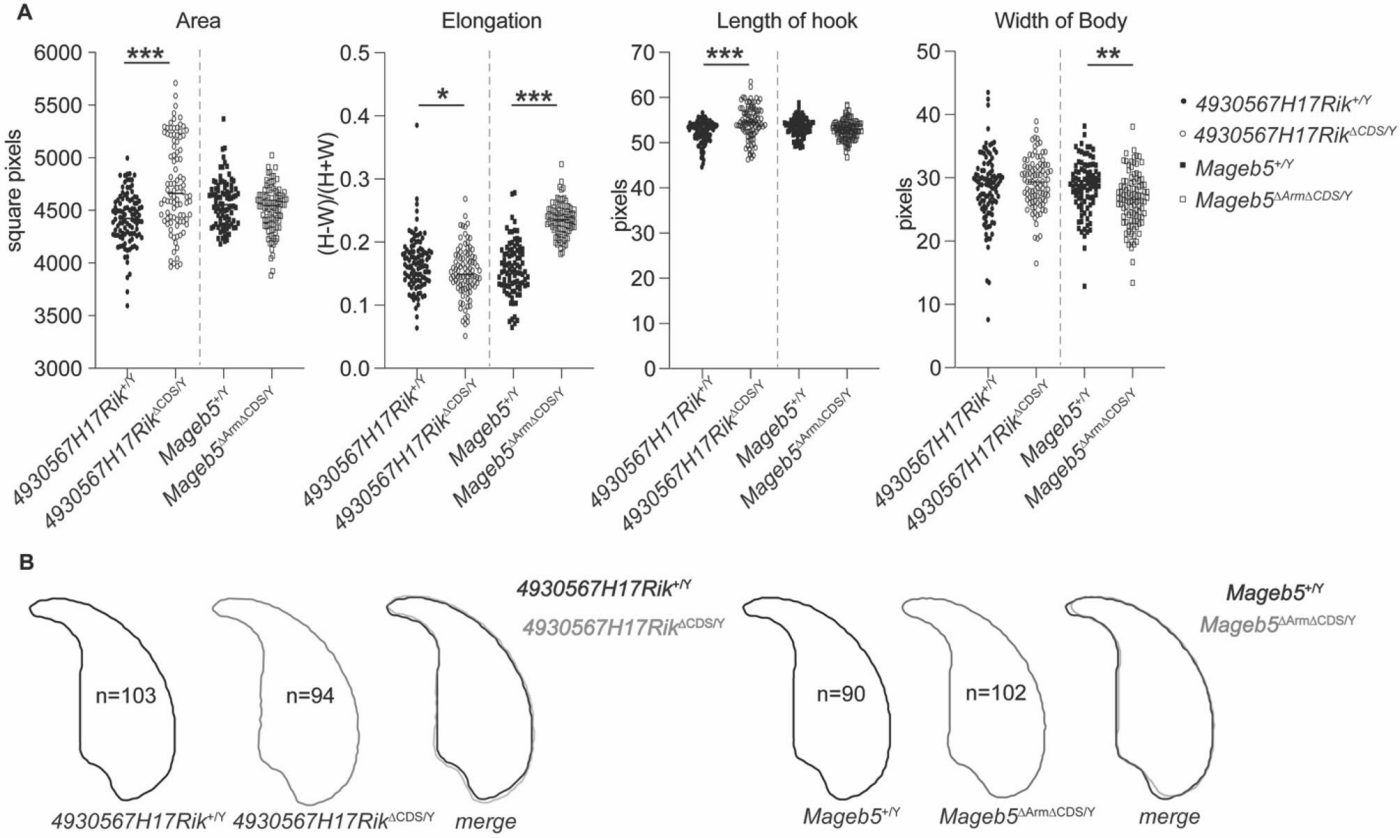
*4930567H17Rik*^∆CDS/Y^ and *Mageb5*^∆Arm∆CDS/Y^ mice display altered sperm morphology. (**A**) Sperm morphology characteristics calculated from assessment of DAPI images processed with a custom plugin to ImageJ^22^. All data were compared using an unpaired two-tailed t-test between *4930567H17Rik*^+/Y^ versus *4930567H17Rik*^∆CDS/Y^ and *Mageb5*^+/Y^ versus *Mageb5*^∆Arm∆CDS/Y^. * p < 0.05 **p < 0.001, ***p < 0.0001. Additional parameters are shown in Fig S7. (**B**) Averages traces and shaded overlays of sperm head morphology from each genotype. Number of sperm assessed are shown inside each respective outline. Representative individual sperm nuclei are also shown in Fig S6B.

Defects in sperm morphology can still be present, despite wild-type levels of sperm production and motility, so we examined sperm morphology in *4930567H17Rik*^∆CDS/Y^ and *Mageb5*^∆Arm∆CDS/Y^ mice. We analyzed multiple attributes of sperm morphology, including area, size of hook, and overall width of the sperm heads, using a custom plugin in ImageJ software^22^. We find that sperm from *Mageb5*^∆Arm∆CDS/Y^ mice were slightly more elongated than *Mageb5*^+/Y^ sperm (0.24 versus 0.16 p =  < 0.0001). Sperm from *4930567H17Rik*^∆CDS/Y^ mice are larger overall (4739 versus 4420 square pixels, p < 0.0001) and have slightly longer hooks (54 versus 52 pixels, p < 0.0001) than *4930567H17Rik*^+/Y^ sperm (Fig. 4A, Fig S6B). The only additional statistical trend observed in morphology was the aspect ratio, the inverse of ellipticity, which was statistically different in *4930567H17Rik*^∆CDS/Y^ (p < 0.02) and in *Mageb5*^∆Arm∆CDS/Y^ mice (p < 0.001) (Fig S6). Despite these differences, 4930567*H17Rik*^∆CDS/Y^ and *Mageb5*^∆Arm∆CDS/Y^ mice are still fertile under laboratory conditions, suggesting that these morphological differences play a minor role in overall fertility.

## Discussion

Our study addresses whether gene families harbored within large singleton X-palindromes are required for male fertility and spermatogenesis in mice. While null mutants of the *Slx* and *Slxl1* gene families harbored in X-palindrome arrays result in male infertility and defects in spermatogenesis^8^, null mutants of the *4930567H17Rik* and *Mageb5* gene families in singleton X-palindromes do not. The absence of an overt reproductive phenotype in male mice lacking *4930567H17Rik* or *Mageb5* may in part be due to genetic redundancy. *4930567H17Rik* is related to heat shock transcription factors, which could have compensating gene family members. Indeed, *Hsf1* and *Hsf2* are expressed in post-meiotic cells^23,24^ and *Hsf2* is known to regulate post-meiotic X- and Y-palindromic gene families^24^ suggesting *Hsf1* and *Hsf2* could compensate for the loss of *4930567H17Rik.* The most likely candidate to compensate for loss of *4930567H17Rik* is *Hsf2*, based on the robust spermatid expression of *Hsf2,* as compared to *Hsf1* (Fig S8, left). Similarly, *Mageb5* has eight X-linked^3^ and one autosomal *Mageb* gene family members expressed in the testis that could potentially compensate for the loss of the *Mageb5* gene family. The most likely candidate to compensate for loss of *Mageb5* is *Mageb3*, because *Mageb3* is expressed at the highest level in spermatids, as compared to other *Mageb* gene family members (Fig. S8, right). To better understand the spermatogenic role of *Mageb5*, and *Mageb* family members, removal of multiple *Mageb* family members, particularly *Mageb3*, may be necessary. For both *4930567H17Rik* and *Mageb5,* further studies investigating these possibly redundant genes could help elucidate the roles of *4930567H17Rik* and *Mageb5* in spermatogenesis. Furthermore, studies of *4930567H17Rik* orthologs in rats or primates*,* that still possesses HSF DNA-binding domains, could shed light on the ancestral function of *4930567H17Rik.*

Despite the lack of overt reproductive phenotypes in *4930567H17Rik*^*∆CDS/Y*^ and *Mageb5*^*∆Arm∆CDS/Y*^ mice, differences in sperm head morphology suggest *4930567H17Rik* and *Mageb5* play a role in sperm development. Sperm head morphology analysis uses DAPI stained images of sperm to detect chromatin. *4930567H17Rik*^+*/Y*^ and *Mageb5*^*∆Arm∆CDS/Y*^ sperm had increased size and elongation, respectively, compared to wild type sperm (Fig. 4). This finding may represent that sperm from these mice have a reduced level of chromatin compaction. Thus, *4930567H17Rik* and *Mageb5* may alter chromatin compaction during spermiogenesis and epididymal transit, a time in development when sperm chromatin compaction is dynamic^25^. Supporting a potential role for *4930567H17Rik* and *Mageb5* in sperm chromatin compaction, both gene families exhibit increasing expression levels from late round spermatids to elongated spermatids (Fig. 1D), a developmental time for sperm chromatin compaction. Tracking the dynamics of sperm head morphology during development and epididymal transit may help further define the role of *4930567H17Rik* and *Mageb5* in sperm development.

Our current analyses of *HSFX* gene families and previous studies on *4930567H17Rik*^16^ demonstrate that *HSFX* and *4930567H17Rik* sequences are rapidly diverging, suggesting the *4930567H17Rik/HSFX* gene family is under positive selection throughout mammals. The gene family’s presence within a X-linked palindrome may facilitate this rapid evolution in multiple ways. First, positive selection is known to be stronger for X-linked genes with male-beneficial functions, because of male sex chromosome hemizygosity^26^. Second, the second gene copy provide more substrate for new beneficial mutations upon which selection pressures can readily act^27^. Third, the second copy could relax constraint on palindromic genes and facilitate the acquisition of novel functions^27^. Fourth, any beneficial mutation arising in one gene copy could be readily spread to other gene copies in the palindrome through arm-to-arm gene conversion^16^. In the future, it will be important to connect how the rapid sequence divergence of *HSFX* and *4930567H17Rik* relates to their spermatogenic functions.

Large palindromic regions are challenging to study in mice and thus are not a priority in large mouse knockout project consortiums^28–31^. CRISPR now enables the study of both X-palindromic structures and their associated gene families. Megabase-sized deletions of arrays of large palindromes demonstrate the necessity of large palindromes and their associated genes for male fertility^8–10^. However, these studies cannot resolve whether the palindrome structure or the associated gene families are responsible for male infertility. Our study demonstrates how CRISPR can generate specific deletions of a single palindrome-associated gene family, while keeping the palindrome structure largely intact. Our study also improves our understanding of large X-palindrome-associated gene function, by demonstrating that individual X-palindrome associated gene families are dispensable for male fertility. Future studies using CRISPR to genetically dissect the importance of palindrome structures versus associated gene families in reproduction will provide a more complete understating of the importance of these large genomic regions and their implications in male fertility.

## Materials and methods

### Generation of mice lacking *4930567H17Rik* and *Mageb5* palindrome-associated gene families

Mice lacking the X-linked palindrome-associated *4930567H17Rik* and *Mageb5* gene families were generated using a CRISPR Cas9 strategy. We selected single guide RNAs (sgRNAs) within the coding sequences of *4930567H17Rik* or *Mageb5* (Table S3). The Cas9 (ESPCAS9PRO, Sigma-Aldrich/Merck KGaA, Darmstadt, Germany) cleavage efficiency of individual sgRNAs was determined via injection of sgRNA (30 ng/ul)/Cas9 (50 ng/ul) complexes into mouse zygotes and screening for edits via PCR using primers flanking sgRNA cut sites (Table S4) and subsequent Sanger sequencing. We selected sgRNAs with cleavage efficiencies of > 30% to delete *4930567H17Rik* and *Mageb5* gene families.

To generate mice lacking the *4930567H17Rik* gene family *(4930567H17Rik*^∆CDS/Y^), C57BL/6 J X SJL hybrid females were crossed with existing *4930567H17Rik*^+/Y^ mice^12^. Zygotes were injected with Cas9 protein (50 ng/μl), a single-stranded oligonucleotide donor (10 ng/μl), and dual sgRNAs (30 ng/μl) targeting each *4930567H17Rik* gene copy to achieve a ~ 650 base pair deletion within each copy on both palindrome arms (Table S3). The deletion breakpoints were verified via PCR and subsequent Sanger sequencing (Fig S7A). An F1 male carrying a deletion of both *4930567H17Rik* coding sequences in cis *(4930567H17Rik*^∆CDS/Y^) was bred to a C57BL/6 J female to generate *4930567H17Rik*^∆CDS/+^ female mice. *4930567H17Rik*^∆CDS/+^ females were backcrossed to C57BL/6 J males to generate *4930567H17Rik*^∆CDS/Y^ mice, which were used for all experiments. *4930567H17Rik*^∆CDS/Y^ mice used in the described experiments were backcrossed to C57BL/6 J for > 7 generations.

To generate mice lacking the *Mageb5* gene family, zygotes from *Mageb5*^∆Arm/+^ females crossed to *Mageb5*^∆Arm/Y^ mice^12^ were injected with Cas9 protein (50 ng/μl), an oligonucleotide donor (10 ng/μl), and dual sgRNAs (30 ng/μl) targeting a ~ 900 bp deletion of *Mageb5* (Table S3). These injections resulted in two independent *Mageb5*^∆Arm∆CDS/Y^ lines, ^“^L1” carrying a 860 bp deletion and “L2” carrying a 400 bp deletion. The deletion breakpoints of the two lines were verified via PCR and Sanger sequencing (Fig S7B). F1 females with both the *Mageb5* palindrome arm and coding sequence deleted in cis were bred to C57BL/6 J males to generate *Mageb5*^∆Arm∆CDS/+^ female mice. *Mageb5*^∆Arm∆CDS/+^ females were backcrossed to C57BL/6 J males for > 10 generations to generate *Mageb5*^∆Arm∆CDS/Y^ mice, which were used for all experiments.

Both *4930567H17Rik*^∆CDS/Y^ and *Mageb5*^∆Arm∆CDS/Y^ mice transmitted the *4930567H17Rik* and *Mageb5* coding sequence deletions through the germline and no changes in overall health were observed due to off-target effects of CRISPR or as a consequence of the deletions. All mice used in these studies were between 3–7 months of age. *4930567H17Rik*^∆CDS/Y^ and *Mageb5*^∆Arm∆CDS/Y^ mice were directly compared to wild type littermates (*4930567H17Rik*^+/Y^ and *Mageb5*^+/Y^ mice) in all experiments allowing for the minimization of the effects of genetic background and age. If wild-type littermates were not available, then age-matched controls were used. Because both *Mageb5*^∆Arm∆CDS/Y L1^ and *Mageb5*^∆Arm∆CDS/Y L2^ mice were able to be maintained easily (had normal breeding), *Mageb5*^∆Arm∆CDS/Y L1^ were used for experiments presented in this work unless otherwise specified. Cages were kept on ventilated racks at 72°F, 30–70% humidity, on a 12 h:12 h light: dark cycle in a specific-pathogen free room. Cages were monitored daily by husbandry personnel and changed every two weeks. Mice were given water and fed Lab Diet 5008 food ad libitum. Adult mice were sacrificed by CO_2_ asphyxiation followed by cervical dislocation and pups were sacrificed by decapitation in compliance with ULAM standard procedures in euthanasia. The Institutional Animal Care and Use Committee of the University of Michigan approved all animal procedures (PRO00009403) and all experiments followed the National Institutes of Health Guidelines of the Care and Use of Experimental Animals and the ARRIVE guidelines.

### Genotyping

Genotypes of *4930567H17Rik*^∆CDS/Y^ and *Mageb5*^∆Arm∆CDS/Y^ mice were determined via PCR on DNA samples collected from 1–2 mm tail snips. Tails were digested in 50 mM NaOH for 20 min at 95 °C and briefly vortexed to dissolve tissues. 50 µl of Tris HCl (pH 6.8) was added to neutralize NaOH and samples were centrifuged at 13,000 rpm for 30 s^32^. PCR was performed with *Taq* DNA polymerase (New England Biolabs) per manufactures instructions. To verify genotypes of *4930567H17Rik*^∆CDS/Y^ and *Mageb5*^∆Arm∆CDS/Y^ mice, we used primers flanking the coding sequence of each gene (primers 1–5 Table S4). For the *Mageb5* lines, we used primers flanking the *Mageb5* palindrome arm to verify the deletion of the palindrome arm, as previously described^12^ (primers 6,7 Table S4).

### Reverse transcriptase-PCR

Total testis RNA was extracted using Trizol (Life Technologies) according to the manufacturer’s instructions. ~ 10 µg of total RNA was DNase treated using Turbo DNase (Life Technologies) and reverse transcribed using Superscript II (Invitrogen) using oligo (dT) primers to generate first-strand cDNA. RT-PCR was performed on adult testis cDNA preparations with primers residing in the single exon coding sequence of *4930567H17Rik* (primers 3,8 Table S4)*,* and with intron-spanning primers for *Mageb5* (primers 9,10 Table S4). Primers to the round spermatid-specific gene *Trim42* (primers 11,12 Table S4) served as a positive control^8^. To control for genomic DNA contamination, a reaction lacking reverse transcriptase was performed in parallel.

### RNA-sequencing

Testis RNA was extracted from three *4930567H17Rik*^∆CDS/Y^ and three *Mageb5*^∆Arm∆CDS/Y L1^ mice, along with three wild-type littermate controls from each line, and DNase treated as described above. Total RNA quality was assessed using the Tapestation 4200 (Agilent) (minimum DV200 value of greater than 30% and a minimum concentration of 3.32 ng/µl). RNA used in this study had RIN (RNA integrity number) values ranging from 6.1–8.9. Ribo-minus (RNaseH-mediated) stranded RNA-seq libraries with indexed adaptors were generated (New England BioLabs). Final libraries were quantitated by Kapa qPCR using Kapa’s library quantification kit for Illumina sequencing platforms (Kapa Biosystems, catalog # KK4835). Pooled libraries were subjected to 150 bp paired-end sequencing according to the manufacturer’s protocol (Illumina NovaSeq6000) giving an average of 50 million reads per sample. Bcl2fastq2 Conversion Software (Illumina) was used to generate de-multiplexed Fastq files. RNA-seq reads were pseudoaligned to the NCBI RefSeq gene annotation for the *Mus musculus* C57BL/6 J (mm10) reference genome by Kallisto^33^, using the default settings. Transcript per million (TPM) numbers were generated by Kallisto. The estimated number of RNA-seq reads aligning to each gene, as provided by Kallisto, were used as input to DESeq^34^ to determine differentially expressed genes between *4930567H17Rik*^∆CDS/Y^ and *Mageb5*^∆Arm∆CDS/Y^ and wild-type mice. All Illumina sequences can be found on NCBI’s sequence read archive under BioProject number: PRJNA748373 and accession numbers of SRR15198217 – SRR15198228.

### Testis histology and staining

Testes were fixed overnight in Bouin’s solution (Ricca Chemical, Arlington, TX). Following fixation, testes were washed in 5–10 ml of 70% ethanol on a rotating tube holder at 4 °C for 6–48 h with three or more changes of ethanol to remove excess Bouin’s. Fixed testes were paraffin embedded, sectioned to 5 μm, and stained with Periodic-Acid Schiff (PAS) and Hematoxylin. Testis sections were imaged on an Olympus BX61 equipped with an Olympus DP73 color camera. Specific germ cell populations were identified by their location within a tubule, nuclear size, and the nuclear staining pattern of chromatin^35^.

### Testis to body weight ratio

To calculate testis/body weight ratio, total testis mass was divided by the total body mass taken at the time of euthanasia.

### Sperm counts, motility and swim-up assay

Following dissection from the body cavity, the two cauda epididymis were dissected and nicked three times to allow sperm to swim out. Cauda were placed in 1 ml of Human Tubal Fluid (HTF) (Millipore) at 37 °C, and rotated in a 37 °C incubator for 10 min. Cauda were removed and a 100 μl aliquot was used for pre-swim-up baseline sperm counts and motility assessment. For swim-up assays, the remaining portion of sperm in HTF was then removed and placed in a new tube using wide bore tips. Sperm were centrifuged for 5 min at 400x*g* and the supernatant discarded. The pellet was re-suspended in 1 ml of fresh 37 °C HTF and centrifuged for 5 min at 400×*g*. The supernatant was removed and 1 ml of fresh 37 °C HTF was carefully overlaid on top of the pellet. The tube was then placed at a 45° angle in a 37 °C incubator for 1 h; after which the top 800 μl containing motile sperm was removed and placed in a new tube. All aliquots of sperm used for counting were diluted 1:10 in H_2_O and counted using a hemocytometer. Counts were performed blind with four technical replicates per mouse. Sperm counts were calculated by taking the average number of sperm from each of the four technical replicates per mouse. Motility was assessed by counting ≈ 200 sperm for each replicate on a hemocytometer across at least 5 frames. Sperm were considered motile if they showed both progressive movement and signs of flagellar activity^36,37^. Percent motility for visual assessment was calculated by dividing the number of motile sperm by the total sperm counted and multiplying by 100. Motility assayed via swim up was calculated from dividing the post swim-up count by the pre-count and multiplying by 100. All analyses between groups were performed with an unpaired two-tailed student’s t-test, unless otherwise noted.

### Fecundity and fertility

Three *4930567H17Rik*^∆CDS/Y^ and three *Mageb5*^∆Arm∆CDS/Y^ mice, and equal numbers of wild-type litter mate controls, were each repeatedly mated with two CD1 females. Litter size was recorded, and the sex of each offspring was determined with sex-genotyping PCR primers (primers13,14 Table S4) specific to the X- and Y-linked gene *Ube1* (Ubiquitin-like modifier activating enzyme, 1 as previously described^8^.

### Sperm head morphology assessment

To assess sperm head morphology, 25 μl of the pre-swim-up sperm aliquot above was placed on a slide and allowed to dry. Cells were fixed for 10 min in 500 μl of 4% PFA (diluted in PBS). Slides were rinsed twice in PBS for 5 min and left to dry. Slides were stained with Vectashield with DAPI (Vector Laboratories) under a 22 × 40 mm cover slip and imaged using an Olympus UPlanSApo 100 × oil objective on an Olympus BX61 equipped with a Hamamatsu Orca-ER camera and Excelitas X-Cite 120LED fluorescence illuminator. Nucleus detection and morphology assessment was performed using the default settings of the custom plugin “Nuclear_Morphology_Analysis_1.20.0_standalone” to the image analysis software ImageJ^22^. ~ 100 sperm heads from each genotype were blindly selected, imaged, and input into the software. Default edge detection settings were used, and sperm heads were manually inspected to ensure all sperm were accurately detected and only sperm were selected by the software. Sperm from *Mageb5*^∆Arm∆CDS/Y^ mice were not originally oriented correctly so the top and bottom vertical border tag was placed manually for the dataset^38^.

## Supplementary Information

Supplementary Information 1.

Supplementary Information 2.

Supplementary Information 3.

## Data Availability

The data underlying sections of this article are available in NCBI’s sequence read archive at https://www.ncbi.nlm.nih.gov/sra and can be accessed under BioProject number: PRJNA748373 and accession numbers of SRR15198217 – SRR15198228. The rest of the data underlying this article are available in the article and in the online supplementary material.
