# Supplementary material for: X-linked palindromic gene families 4930567H17Rik and Mageb5 are dispensable for male mouse fertility
Source: Sci Rep. 2022 May 20;12:8554. doi: 10.1038/s41598-022-12433-9 (PMC9122934; doi:10.1038/s41598-022-12433-9)
Supplement: Supplementary file 1 — Supplementary Information 1. [file 41598_2022_12433_MOESM1_ESM.docx]

Supplementary Information

**X-linked palindromic gene families *4930567H17Rik* and *Mageb5* are dispensable for male mouse fertility**

Evan R. Stark-Dykema^1,2^, Eden A. Dulka^1,2^, Emma R. Gerlinger^1^, Jacob L. Mueller^1,*^

^1^ Department of Human Genetics, University of Michigan Medical School, Ann Arbor, MI 48109, USA

^2^ Co-authors

^*^Correspondence: E-mail: [jacobmu@umich.edu](mailto:jacobmu@umich.edu)

**
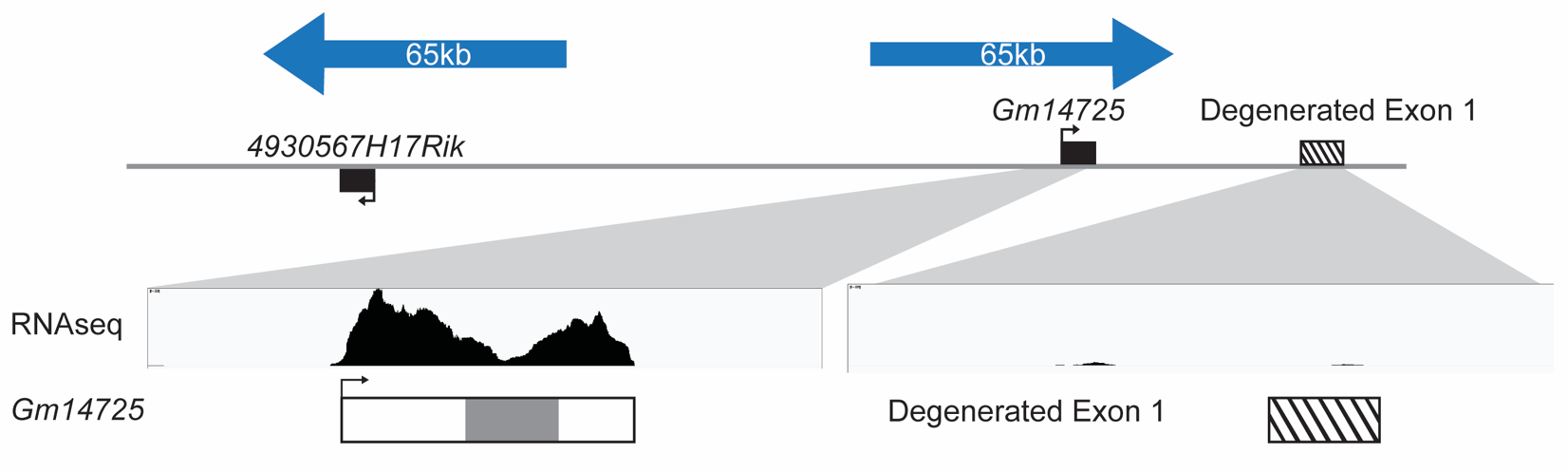
**

**Figure S1. Pseudogenization of a single exon of *4930567H17RIK* in mice.**

Top: RNA-seq data showing lack of transcription of the ancestral HSFX first exon (Degenerated Exon 1) in mice. Bottom: Aligned representation of *Gm14725* (left) and the ancestral first exon (right), dark grey shading is a glutamic acid repeat region.


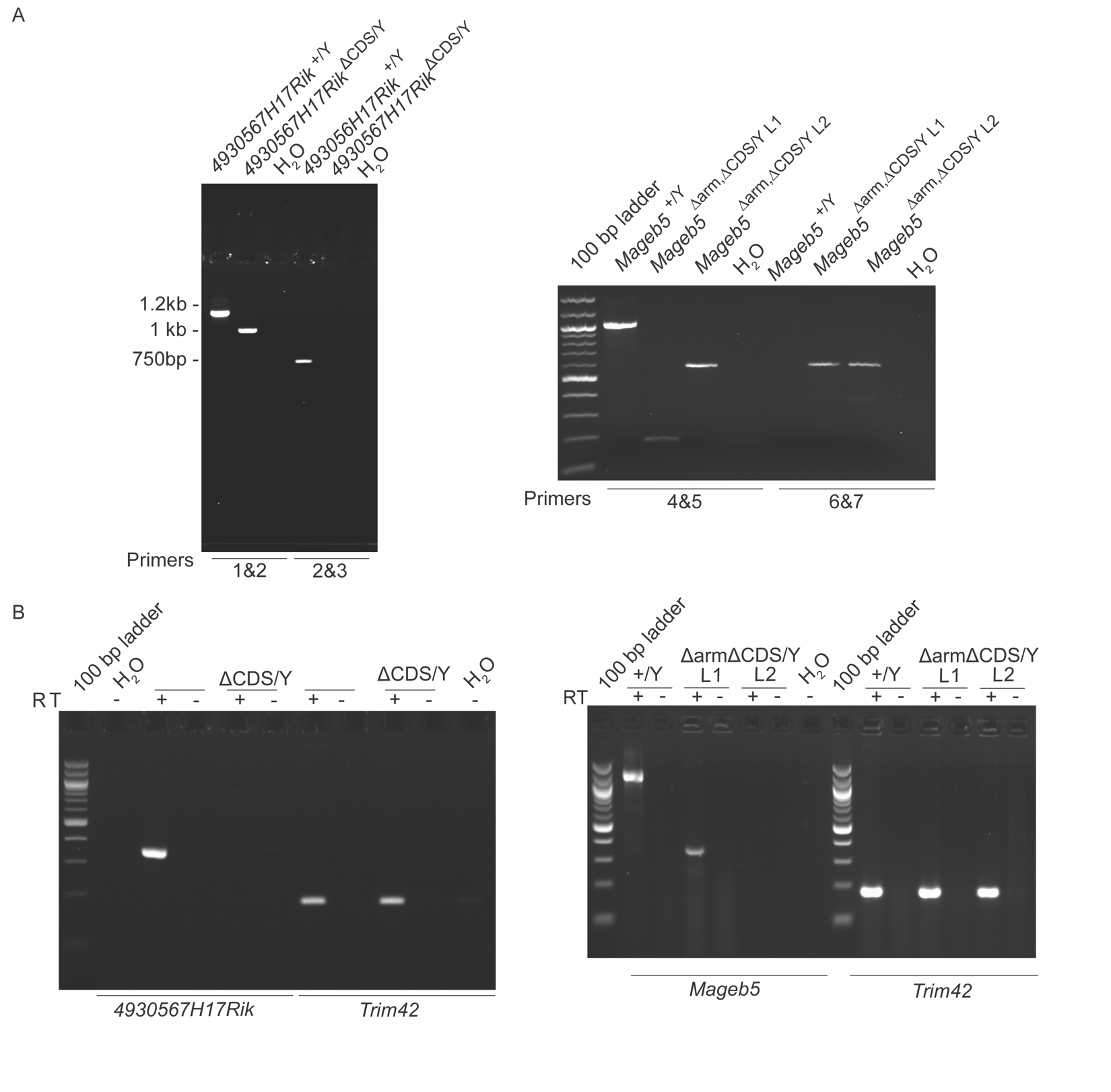


**Figure S2. Full-size gel images of DNA and RNA validation of *4930567H17Rik*^∆CDS/Y^, and *Mageb5*^∆Arm∆CDS/Y^ mice.** (A) PCR genotyping of DNA from mutant and wild type *4930567H17Rik* and *Mageb5* mice shown in Figure 2B. Numbered primers correspond to panel A in Figure 2. (B) Gel images corresponding to Figure 2C. RT-PCR of *4930567H17Rik* and *Mageb5* cDNA from one *4930567H17Rik* line and *Mageb5* L1 and L2.

**
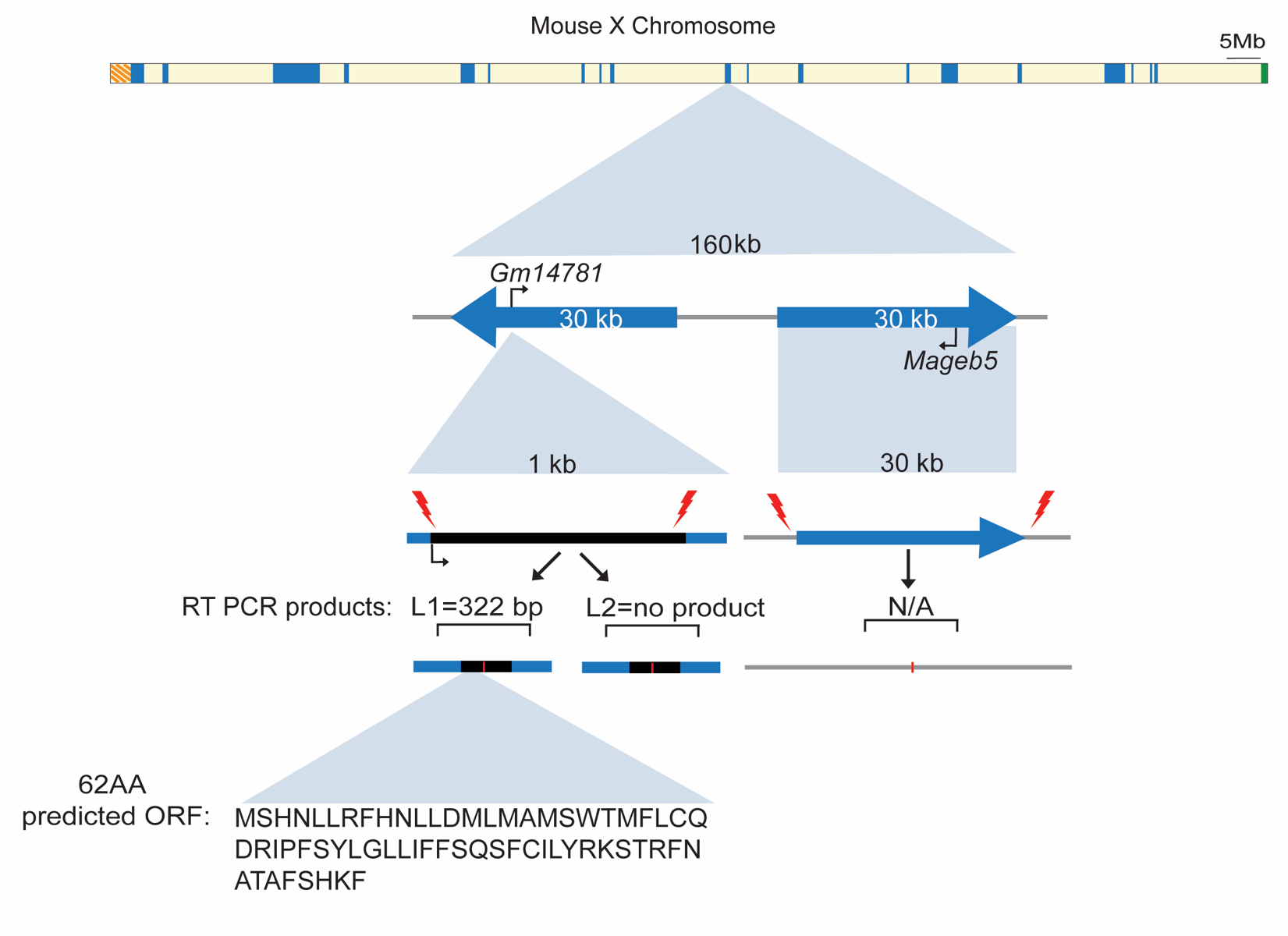
**

**Figure S3. RT-PCR analysis and predicted ORFs in *Mageb5*^Δarm, ΔCDS/Y^ mice.**

**
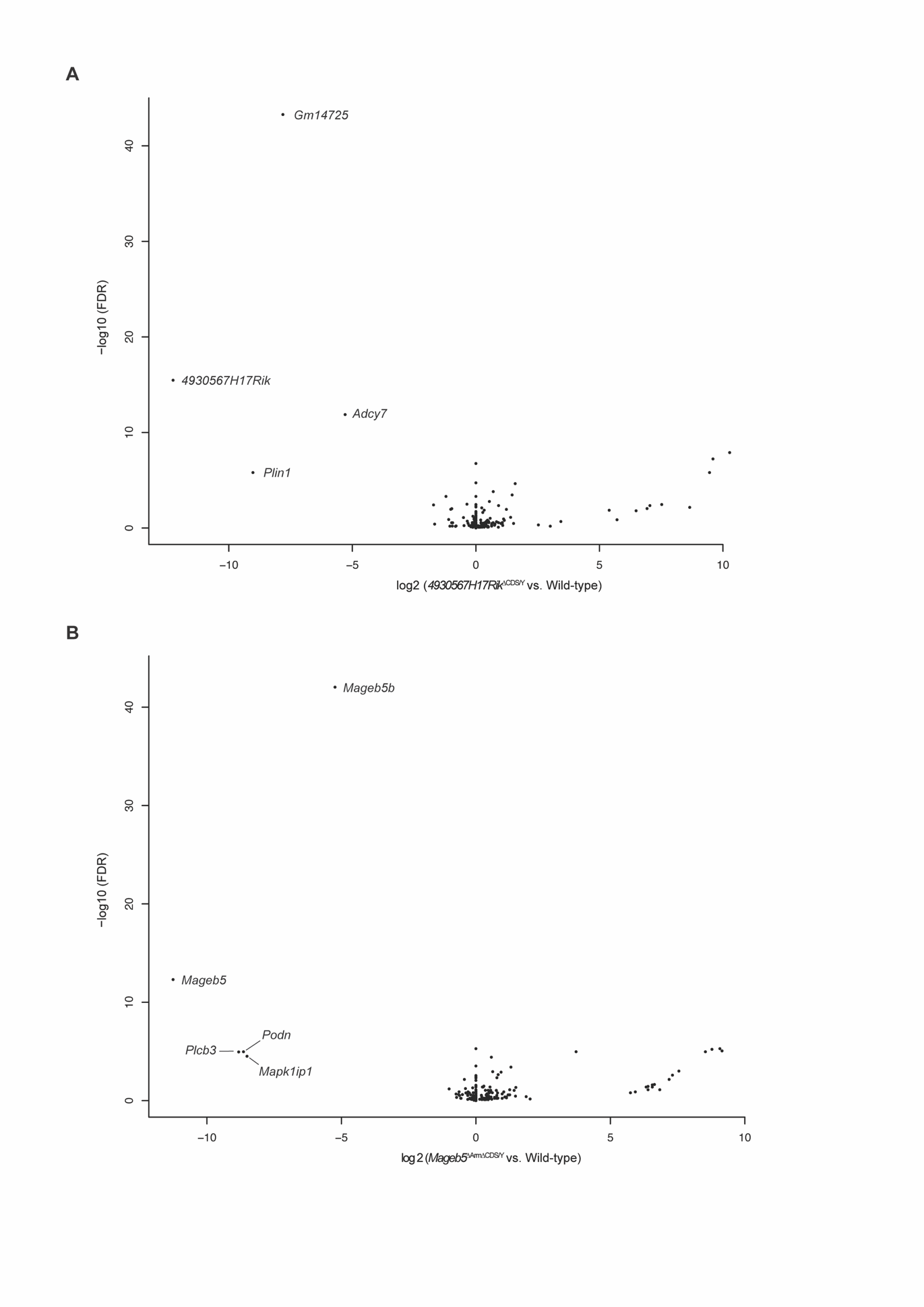
**

**Figure S4. Differential expression of genes in testes of *4930567H17Rik*^∆CDS/Y^ and *Mageb5*^∆Arm∆CDS/Y^ mice.** Volcano plots of differentially expressed genes (p-value <0.0001) from whole testis RNA-seq in *4930567H17Rik*^∆CDS/Y^ (panel A) or *Mageb5*^∆Arm∆CDS/Y^ (panel B) mice and their wild-type littermate controls.

**
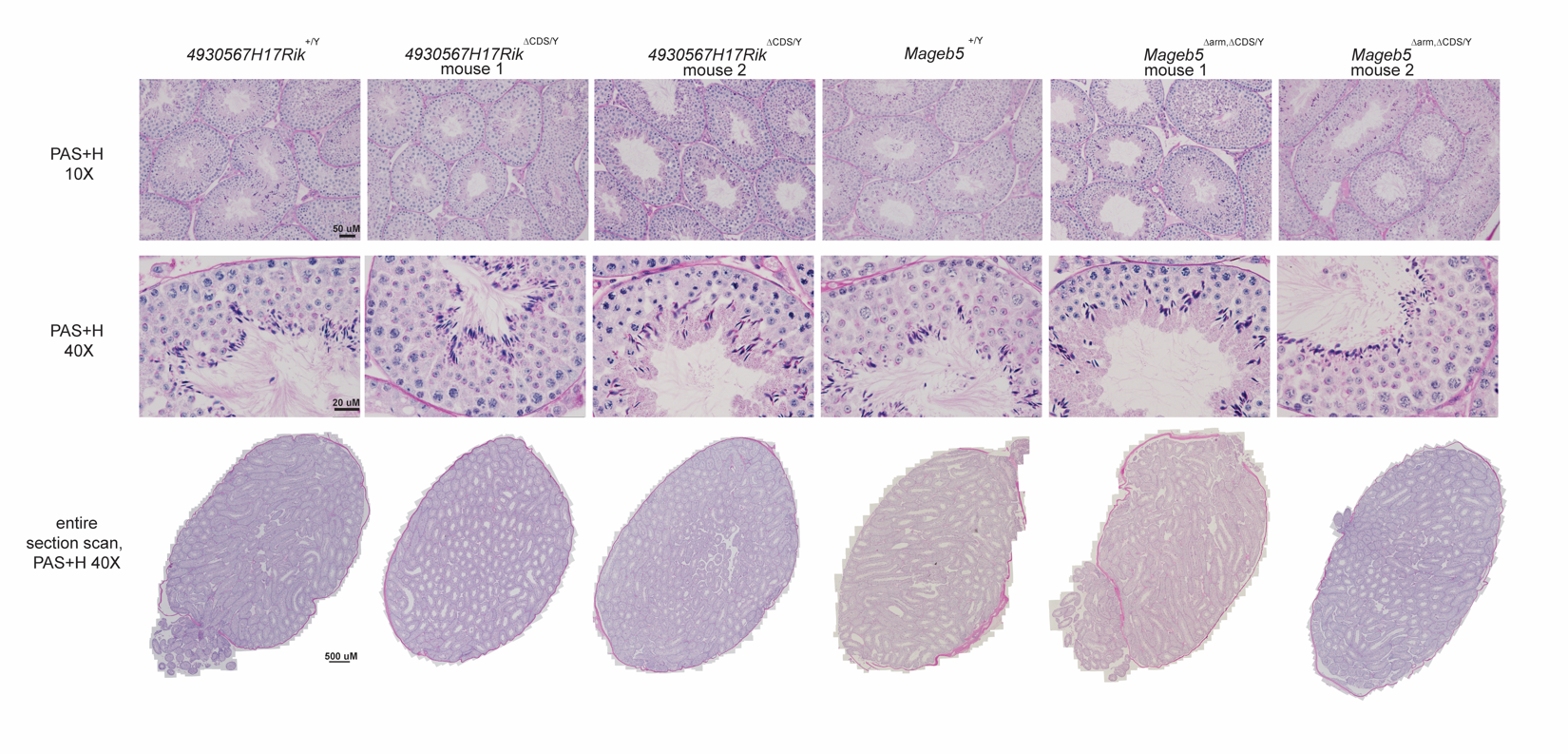
**

**Figure S5. Histological sections and whole section scans from *4930567H17Rik*^∆CDS/Y^ and *Mageb5*^∆Arm∆CDS/Y L1^ mouse testes do not exhibit overt differences spermatogenic cell populations.** Sections were stained with periodic acid-Schiff and hematoxylin (PAS+H). Top row: 10X magnification, multiple tubules are displayed. Middle row: representative tubule from each individual used in the top row. Bottom row: scan of the entire testis section from each mouse.

**
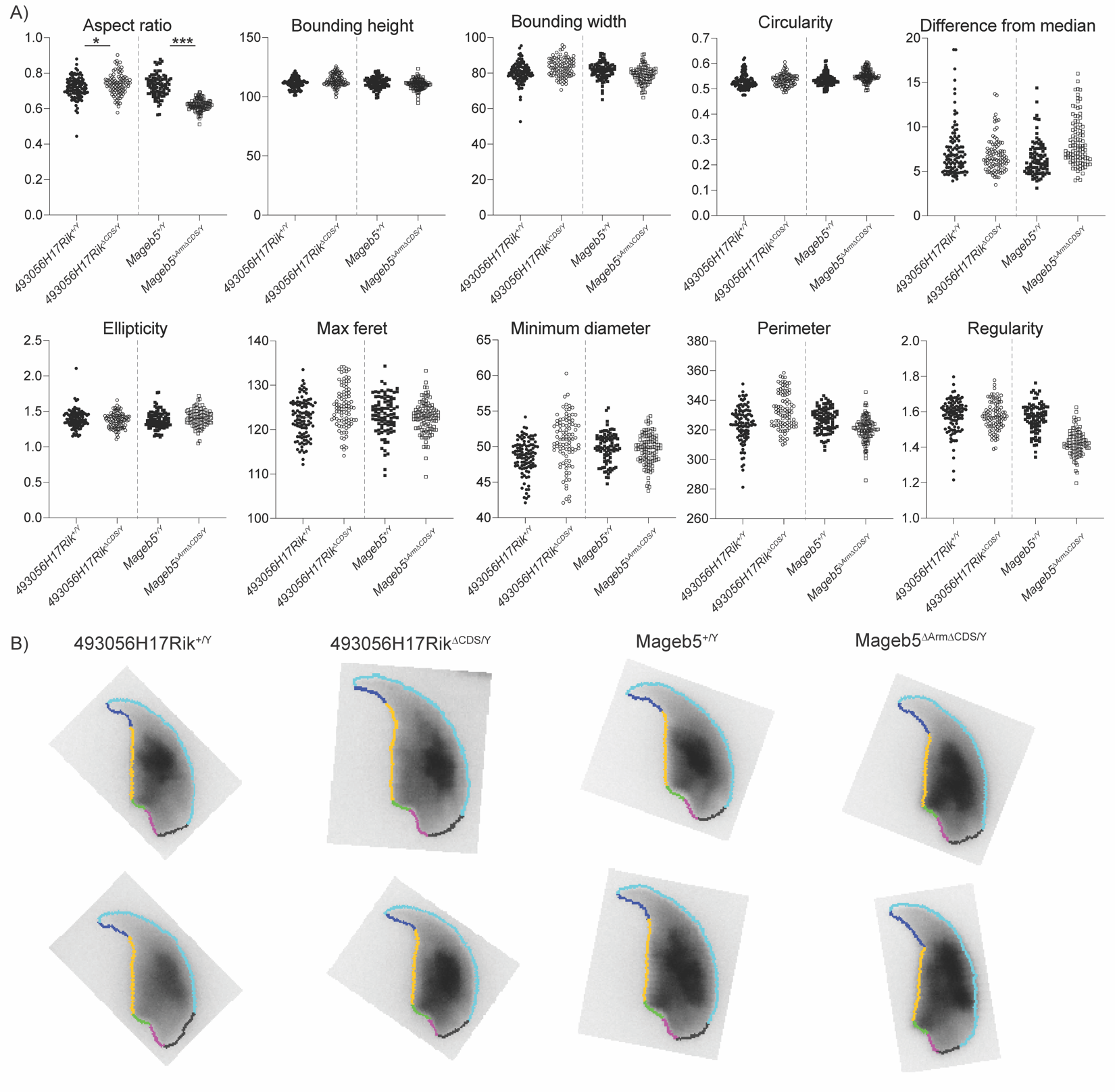
**

**Figure S6. Additional sperm morphology assessment parameters for *4930567H17Rik*^∆CDS/Y^ and *Mageb5*^∆Arm∆CDS/Y^ mice.** A) Sperm characteristics calculated from assessment of DAPI images processed with a custom plugin to ImageJ from Skinner et al, 2019. * p<0.05 **p<0.001 , ***p< 0.0001. B) Representative images of sperm heads taken during analysis. Colors represent different segments defined by the plugin used during for calculations.


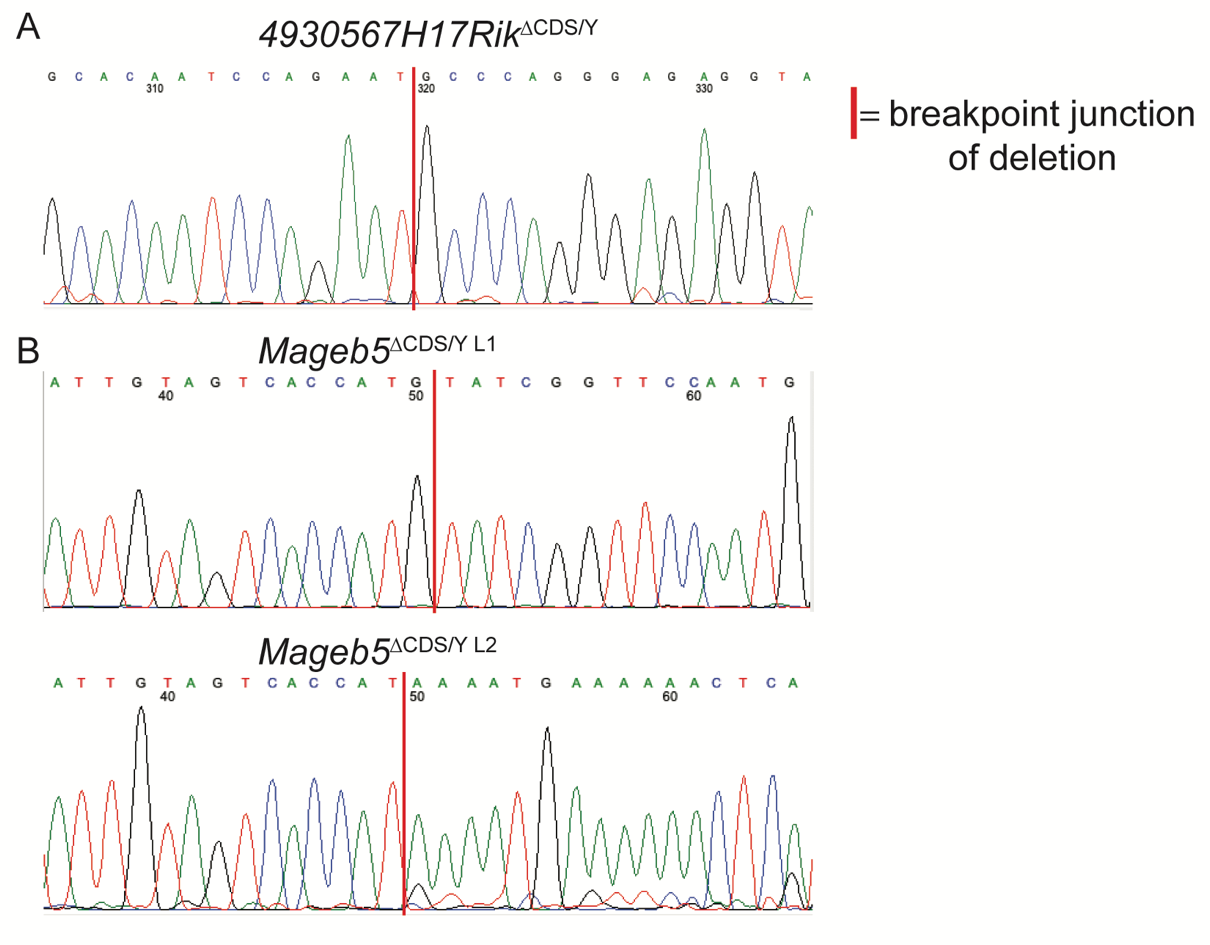


**Figure S7. Deletion breakpoints in coding sequences of *4930567H17Rik* and *Mageb5* gene families*.*** Sanger sequencing results derived from PCR products showing breakpoints in *4930567H17Rik*^∆CDS/Y^ *Mageb5*^∆Arm∆CDS/Y L1^ and *Mageb5*^∆Arm∆CDS/Y L2^ mice.

**
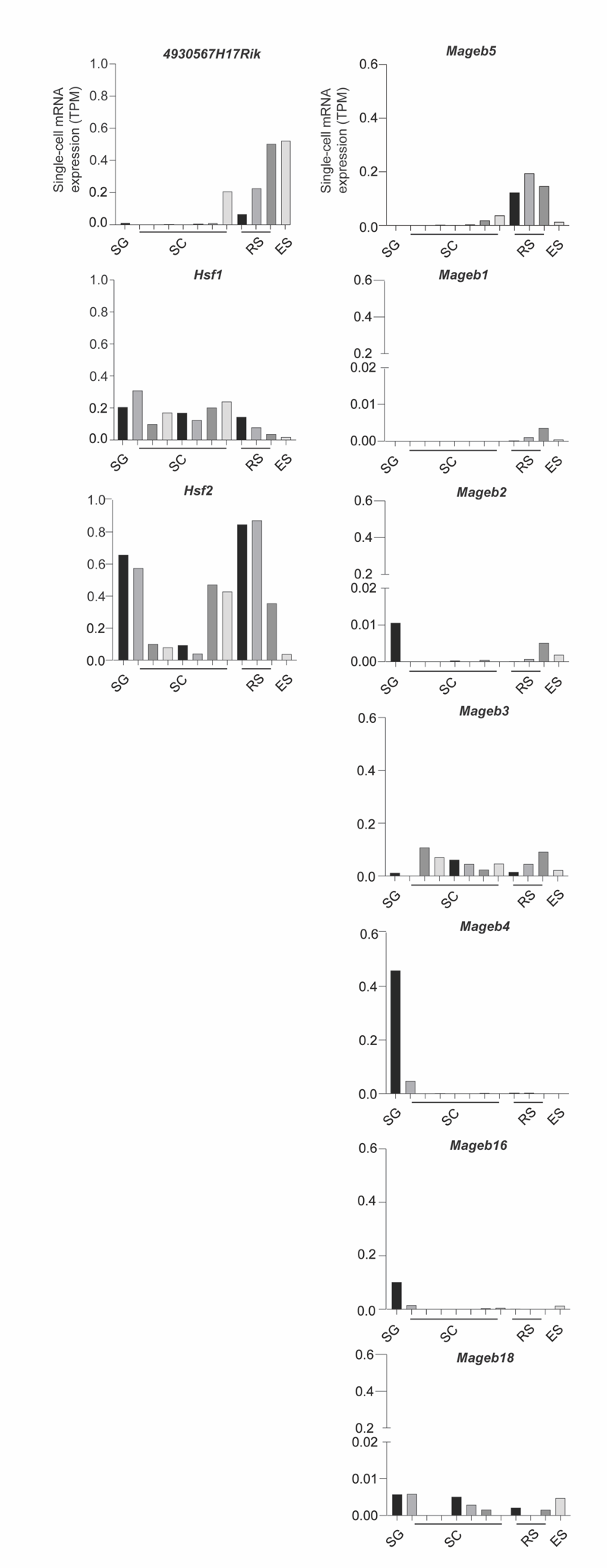
**

**Figure S8. Expression of genes related to *4930567H17Rik* and *Mageb5* in spermatogenic cells.** Natural log values of UMI counts from single-cell RNA-seq data for *4930567H17Rik* and *Mageb5* from Green, et. al.^19^.

**Table S3.** sgRNA sequences used to generate *4930567H17Rik*^∆CDS/Y^ and *Mageb5*^∆Arm∆CDS/Y^ mice*

| **Region** | **Sequence** |
| --- | --- |
| 493Rik_gRNA_1 | CTGCACAATCCAGAATAGAC |
| 493Rik_gRNA_2 | TCCGTATGCT CTTCAGCCCA |
| 493Rik_CDSdel  _ssODN | GAAAAAGGTAGTTATTCTCAGGCAGTACCTCTCCCTGGGCATTCTGGATTGTGCA  GCATTAAACATAAATCAAGACATTC |
| Mageb5_gRNA_1 | TTGTAGTCACCATGCCC |
| Mageb5_gRNA_2 | CATTGGAACCGATAGCA |
| Mageb5_CDSdel  _ssODN | TACTAGGTTCAACGCCACTGCCTTTAGCCATAAATTCTAGGACTTTCATCTTGGT  AGTTTTAAGTAGAAGTA |

*****Sequences used for arm deletions of the other gene copies of *4930567H17Rik* and *Mageb5* previously published in [1]

**Table S4.** Primers and sequences used to verify *4930567H17Rik*^∆CDS/Y^ and *Mageb5*^∆Arm∆CDS/Y^ mice and sex of pups for sex-ratio assays

| **Figure : Primer number** | **Primer name** | **Sequence** |
| --- | --- | --- |
| 2A:1 | 493Rik_CDS_5’F | CCACCTCTTGAGGAATGGAA |
| 2A:2 | 493Rik_CDS_3’R | CAGGCAAGGAGGAGTGAGTC |
| 2A,C:3 | 4930567H17Rik_R | TCTGCATGGGTCGTATGA |
| 2A:4 | Mageb5_CDS F | TTGCCCTCTCATTATCTCCTACA |
| 2A:5 | Mageb5_CDS R | GCCACTGCCTTTAGCCATAA |
| 2A:6 | Mageb5_Arm F | GTTTGCAGAGTTGTGGACTGATAC |
| 2A:7 | Mageb5_Arm R | TGCATTGATCAAAAGGGAGA |
| 2C:8 | 4930567H17Rik_F | GGGCCTCTGAGACCACAT |
| 2C:9 | Mageb5 intron F | GGGAGAATCATCCACTTCTGA |
| 2C:10 | Mageb5 3' UTR | AAATCTCAAACGTGATGTTATAATTTC |
| 2C:11 | Trim42 F | GACTGTCTCAAGGCCTTC |
| 2C:12 | Trim42 R | CATGGCCATTGTGGAAAG |
| 13 | Ube1XY_F | TGGATGGTGTGGCCAATG |
| 14 | Ube1XY_R | CACCTGCACGTTGCCCTT |
